# Antibody Responses and the Vaccine Efficacy of Recombinant Glycosyltransferase and Nicastrin Against *Schistosoma japonicum*

**DOI:** 10.3390/pathogens14010070

**Published:** 2025-01-14

**Authors:** Bowen Dong, Haoran Zhong, Danlin Zhu, Luobin Wu, Jinming Wang, Hao Li, Yamei Jin

**Affiliations:** 1 National Reference Laboratory for Animal Schistosomiasis, Key Laboratory of Animal Parasitology of Ministry of Agriculture and Rural Affairs, Shanghai Veterinary Research Institute, Chinese Academy of Agricultural Sciences, Shanghai 200241, China; dongbw131420@163.com (B.D.); haoranzhong@shvri.ac.cn (H.Z.); 13957236028@163.com (D.Z.); lihao@shvri.ac.cn (H.L.); 2 College of Life Sciences, Shanghai Normal University, Shanghai 200234, China; luobin.wu@foxmail.com; 3 State Key Laboratory for Animal Disease Control and Prevention, College of Veterinary Medicine, Lanzhou University, Lanzhou Veterinary Research Institute, Chinese Academy of Agricultural Sciences, Lanzhou 730000, China; wjm0403@caas.cn; 4 Key Laboratory of Veterinary Parasitology of Gansu Province, Gansu Province Research Center for Basic Disciplines of Pathogen Biology, Lanzhou 730046, China

**Keywords:** *Schistosoma japonicum*, SjGT, SjNCSTN, immune protection

## Abstract

Schistosomiasis is a neglected tropical disease and the second most common parasitic disease after malaria. While praziquantel remains the primary treatment, concerns about drug resistance highlight the urgent need for new drugs and effective vaccines to achieve sustainable control. Previous proteomic studies from our group revealed that the expression of *Schistosoma japonicum* glycosyltransferase and nicastrin as proteins was higher in single-sex males than mated males, suggesting their critical roles in parasite reproduction and their potential as vaccine candidates. In this study, bioinformatic tools were employed to analyze the structural and functional properties of these proteins, including their signal peptide regions, transmembrane domains, tertiary structures, and protein interaction networks. Recombinant forms of glycosyltransferase and nicastrin were expressed and purified, followed by immunization experiments in BALB/c mice. Immunized mice exhibited significantly elevated specific IgG antibody levels after three immunizations compared to adjuvant and PBS controls. Furthermore, immunization with recombinant glycosyltransferase and nicastrin significantly reduced the reproductive capacity of female worms and liver egg burden, though egg hatchability and adult worm survival were unaffected. These findings demonstrate that recombinant glycosyltransferase and nicastrin are immunogenic and reduce female worm fecundity, supporting their potential as vaccine candidates against schistosomiasis.

## 1. Introduction

Schistosomiasis, caused by trematode worms of the *Schistosoma* genus, is a parasitic disease that poses a global health threat, second only to malaria in its impact [1]. Over 250 million people are infected globally, with more than 780 million individuals at risk across 78 endemic countries. The disease imposes substantial public health and economic burdens, particularly in rural and impoverished regions, where it reduces productivity, impairs development in children, and incurs significant healthcare costs [2]. Its pathogenesis is primarily driven by the formation of granulomas and secondary necrosis, being triggered by the deposition of schistosome eggs in various tissues and organs [3], resulting in severe complications, including ascites and variceal hemorrhage, which may ultimately prove fatal [4,5]. Despite being the primary treatment option, praziquantel does not prevent reinfection [6], and concerns about potential drug resistance further complicate disease control efforts [7]. Although effective vaccines have been developed for other parasitic infections, such as hookworms [8], no vaccines are currently available for schistosomiasis. Thus, the urgent need for innovative drugs and vaccines to combat schistosomiasis remains a critical priority [9].

Proteomic studies conducted by our team on single-sex and bisexual infected male *Schistosoma japonicum* revealed that the expression levels of glycosyltransferase (GT) and nicastrin (NCSTN) were significantly higher in single-sex infected males compared to mated males. This differential expression is likely linked to the development of male schistosomes and the transmission of signals from males to regulate the growth and reproduction of females [10]. GT, a universally produced enzyme across species [11,12], plays a key role in synthesizing the sugar components of disaccharides, glycans, glycosides, and complex glucans [13]. Schistosomes, in particular, produce a variety of complex glycans and glycoproteins that interact with the host’s innate and adaptive immune systems [14]. NCSTN, on the other hand, is an integral component of the transmembrane γ-secretase protein complex. Levitan et al. [15] found that NCSTN, as a homologue of Aph-2, regulates worm fertility through the LIN-12/Notch signaling pathway. Additionally, NCSTN interacts with Notch receptor proteins and Aβ A4 precursor proteins, underscoring its role in reproductive and developmental processes [16]. Based on the experimental results, both GT and NCSTN appear to be involved in various biological processes and may play a critical role in the reproductive development of male schistosomes. However, their precise biological functions in the reproductive development of *S. japonicum* and their potential as immunological targets remain to be fully explored.

In this study, the potential biological functions of the SjGT and SjNCSTN proteins were investigated through analyses of their signal peptide regions, structural domains, tertiary structures, and potential protein interaction networks. Additionally, recombinant SjGT (rSjGT) and SjNCSTN (rSjNCSTN) were constructed and evaluated for their potential as immunogens against schistosomiasis, providing valuable insights for vaccine development. Notably, our study demonstrated that immunizing mice with a protein highly expressed in male *S. japonicum* also impacts the reproductive development of female worms, highlighting a novel strategy for targeting parasite fecundity through vaccination.

## 2. Materials and Methods

### 2.1. Parasite

Six-week-old BALB/c male mice, purchased from the Shanghai Laboratory Animal, Co., Ltd. (Shanghai, China), were used for worm collection. The *S. japonicum* Anhui strain was provided by the Shanghai Veterinary Research Institute, Chinese Academy of Agricultural Sciences (Shanghai, China). The numbers of viable cercariae were determined prior to infection using a light microscope. Worms were collected by hepatic portal perfusion. The protocols of all animal experiments were approved by the Animal Care and Use Committee of Shanghai Veterinary Research Institute, Chinese Academy of Agricultural Sciences (Shanghai, China; approval no: SV-20230818-02).

### 2.2. Bioinformatics Analysis of SjGT and SjNCSTN

The molecular characteristics of SjGT (Genbank: KAH8853798.1) and SjNCSTN (GenBank: KAH8853349.1) were analyzed by a bioinformatic method. The signal peptide was predicted using the protein structure analysis platform of SignalP (version 4.1) [17]. Transmembrane regions were predicted using the protein structure analysis platform of Detai Biology (http://www.detaibio.com/tools/, accessed on 20 March 2024). Protein tertiary structures were predicted using the SWISS-MODEL [18]. Amino acid (aa) sequences were used as queries to identify SjGT and SjNCSTN homologues. Jalview software (version 2.11.4.1) was used to compare protein sequences with sufficient similarities between different species [19]. The protein interaction was analyzed by STRING (version 12.0) [20]. The expression data of SmNCSTN (GenBank no: XP_018648822.1, also referred to as Smp_167240, obtained from WormBase ParaSite, version WBPS19) [21] in a publicly RNAseq dataset (version 7) [22] and a single-cell RNAseq dataset [23] of *Schistosoma mansoni*, were taken as a reference for the expression of SjNCSTN. A heatmap representing the Smp_167240 gene expression throughout the life stages (egg, miracidium, sporocyst, cercaria, schistosomulum, 21-day juvenile, 28-day juvenile, 35-day adult, 38-day adult, adult older than 42 daya) of MM and MF was constructed.

### 2.3. Cloning of SjGT and SjNCSTN

Total RNA was extracted from adult *S. japonicum* worms using TRNzol reagent (TianGen, Beijing, China), and cDNA was reverse transcribed from total RNA with the Hifair II 1 st Strand cDNA Synthesis Kit (Yeasen, Shanghai, China). The primers and enzyme restriction sites are shown in Supplementary Table S1. PCR was performed with the following cycling conditions: 94 °C for 3 min; 30 cycles at 94 °C for 30 s, 56 °C for 45 s, and 72 °C for 45 s; followed by 72 °C for 5 min. Then, the cDNA product was cloned into the pGEM T-easy vector and sequenced.

### 2.4. Protein Expression and Purification

The full-length cDNA of SjGT and SjNCSTN were subcloned into the pET-28a(+) vector using the restriction enzymes *EcoRI*/*BamHI* and *XhoI* to produce a protein that constructed recombinant pET-28a-SjGT and pET-28a-SjNCSTN plasmids, which were transformed into *Escherichia coli* BL21 (DE3) cells (Invitrogen, Waltham, MA, USA), and recombinant clones were obtained by antibiotic selection. The recombinant SjGT and SjNCSTN proteins were then overexpressed in the presence of isopropyl-b-d-thiogalac-topyranoside (Yeasen, Shanghai, China). Next, the transformed cells were grown in Luria broth (LB) plus kanamycin (1 mg/mL) (Yeasen, Shanghai, China) at 37 °C until OD values of 0.7 were read at 600 nm using a microplate reader (BioTek, Winooski, VT, USA), and IPTG was added to the culture at a final concentration of 1 mM. After 0–8 h of induction, cells were harvested, the expression of rSjGT and rSjNCSTN proteins was analyzed by SDS-PAGE, and the histidine-tagged fusion rSjGT and SjNCSTN protein was then purified from *E.coli* lysates by metal affinity chromatography using Ni-NTA His Bind Resin Chromatography (Novagen, Beijing, China). The concentrations of the purified proteins were determined by the Bradford method using bovine serum albumin (Yeasen, Shanghai, China) as the standard [24].

### 2.5. Western Blot Analysis

Briefly, the purified protein was separated using 5% stacking gels and 12% resolving gels, then transferred onto 0.45 μm nitrocellulose membranes (Yeasen, Shanghai, China). The membranes were blocked with 5% non-fat milk (Yeasen, Shanghai, China) in PBS for 2 h at room temperature and icubated overnight at 4 °C with rabbit polyclonal anti-adult worm proteins of *S. japonicum* (preserved in our lab). After washing three times with PBS-Tween 20 (PBST) (Yeasen, Shanghai, China), the membranes were probed with horseradish peroxidase (HRP)-conjugated anti-rabbit IgG antibody (Invitrogen, Waltham, MA, USA) for 2 h. After another three washes for 5 min each with PBST, the gray color was visualized using ChemiSignal Plus ECL (Yeasen, Shanghai, China).

### 2.6. Vaccination of Mice with Recombinant SjGT and SjNCSTN

The 206 adjuvant (Seppic, Paris, France) was used according to the manufacturer’s instructions. Male BALB/c mice aged 6–8 weeks were randomly divided into 4 groups (rSjGT-immunized, rSjNCSTN-immunized, 206-adjuvanted, and PBS groups, 10 mice each). rSjGT or rSjNCSTN was mixed with 206 adjuvant at a ratio of 46:54 and injected subcutaneously into the immunized mice three times at 2-week intervals, with the first injection of 50 μg/100 μL/mouse and the next two injections of 25 μg/100 μL/mouse, while the 206-adjuvanted and PBS groups were injected with an equal volume of an ultrasonically emulsified mixture of 206 adjuvant or PBS solution (Yeasen, Shanghai, China). Two weeks after the third immunization, all mice were percutaneously infected with 40 ± 2 cercaria. At 42-day post-infection (dpi), the worms were collected and counted, as previously described [25].

### 2.7. Enzyme-Linked Immunosorbent Assays (ELISA) Using Sera from Immunized Mice

At 42 dpi, blood samples were collected from mice in each group by retro-orbital bleeding. Specific IgG antibodies against rSjGT and rSjNCSTN were detected by ELISA. 96-well microtiter plates were coated (Costar, New York, NY, USA) overnight at 4 °C with 100 μL of soluble rSjGT or rSjNCSTN (10 μg/mL) diluted in carbonate–bicarbonate buffer (pH = 9.6) (Yeasen, Shanghai, China). The plates were washed three times using TBST (0.05% Tween 20 in PBS) (Yeasen, Shanghai, China), then blocked with 3% BSA in TBST for 1 h at 37 °C. After washing three times, all test sera were diluted 1:100 with TBST and incubated at 100 μL/well for 2 h at 37 °C. The plates were then washed three times, and goat anti-mouse IgG (1:200 dilution, Sigma, Waltham, MA, USA) was conjugated to horseradish peroxidase in TBST and was added to the wells (100 μL/well). After 1 h incubation at 37 °C, the plates were washed three times and 100 μL of 3,3′ 5,5′ -tetramethyl benzidine dihydrochloride (Yeasen, China) was added to each well. The reaction was incubated for 10 min at 37 °C in the dark and stopped using 2 M sulfuric acid (50 μL/well) (Yeasen, Shanghai, China). The OD values were read at 450 nm using a microplate reader (BioTek, Winooski, VT, USA).

### 2.8. Worm Burden, Liver Egg Count, and Hatching Rate

Mice were sacrificed at 42 dpi. Worms were collected in sterile mesh via hepatic portal vein perfusion and counted. The livers were weighed and homogenized with PBS (pH = 7.4) and filled to 20 mL. Then, 5 mL of the homogenate was then taken and an equal volume of 10% NaOH (Sangon biotech, Shanghai, China) was added. After digestion at 37 °C for 1 h with 10% NaOH, the eggs were counted under a microscope. For the hatching of eggs, 4 mL of the above homogenate was transferred to a long-neck flask, diluted with an appropriate amount of dechlorinated water, and incubated for 6 h at 25 °C in daylight; the miracidia were stained with an iodine tincture and then the miracidia were counted under the microscope. The number of eggs per gram of liver, the number of eggs per female worm, and the hatchability were calculated as previously described [25].

### 2.9. Statistical Analysis

All statistical analyses were conducted using GraphPad Prism 8.0.1 Software. All the statistically significant differences among more than two groups were executed by one-way analysis of variance a with post hoc Tukey’s test. Data are expressed as the mean ± standard deviation (SD). A probability (*p*) value of ≤0.05 was considered statistically significant. “*” represents *p* < 0.05 and“**” represents *p* < 0.01.

## 3. Results

### 3.1. In Silico Characterization

The signaling peptides and transmembrane regions of SjGT and SjNCSTN were analyzed to clarify the biological functions of these proteins. The results revealed that SjGT lacked a signaling peptide (Supplementary Figure S1A) and transmembrane region (Supplementary Figure S1B), while SjNCSTN possessed a signaling peptide at aa residues 1–23 (Supplementary Figure S1C) and a transmembrane region at aa 658–680 (Supplementary Figure S1D).

### 3.2. Homology Analysis of SjGT and SjNCSTN

Homology analysis of SjGT against the GT proteins of *S. mansoni* (XP_018648185.1), *Schistosoma haematobium* (XP_051073755.1), *Homo sapiens* (KAI2568696.1), and *Mus musculus* (XP_030110433.1) showed that the sequence identities were 80%, 82%, 27%, and 35%, respectively (Supplementary Figure S2A). Meanwhile, homology analysis of SjNCSTN against the NCSTN proteins of *S. mansoni* (XP_018648822.1), *S. haematobium* (CAH8456786.1), *H. sapiens* (KAI2519972.1), and *M. musculus* (BAE41750.1) showed that the sequence identities were 75%, 77%, 30%, and 27%, respectively (Supplementary Figure S2B).

### 3.3. Phylogenetic Analysis of SjGT and SjNCSTN

The phylogenetic analysis of SjGT and SjNCSTN revealed that human and mouse GT and NCSTN were positioned on distinct branches from those of schistosomes, indicating distant evolutionary relationships. The GT protein of *S. mansoni* clustered on the same branch as SjGT but was separate from the GT of *S. haematobium* (Supplementary Figure S3A). Meanwhile, SjNCSTN was located on the same branch of the NCSTN protein of *S. haematobium* but on a different branch from *S. mansoni* (Supplementary Figure S3B).

### 3.4. Predicted Protein Structures of SjGT and SjNCSTN

**Figure 1:**
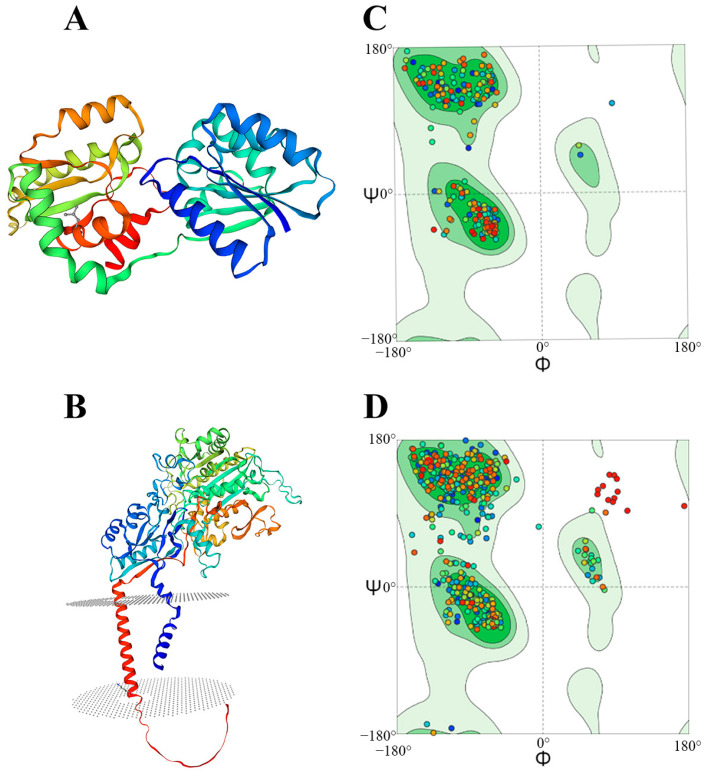
Protein structure prediction of SjGT and SjNCSTN. (**A**) A 3D model of SjGT. (**B**) A 3D model of SjNCSTN. (**C**) A Ramachandran plot analysis of SjGT. (**D**) A Ramachandran plot analysis of SjNCSTN.

The tertiary structures of SjGT and SjNCSTN were constructed using 3D models (Figure 1A,B). The global quality estimation scores of SjGT and SjNCSTN were 0.91 and 0.86, respectively, suggesting a high level of confidence in the 3D models. In addition, a Ramachandran plot analysis showed that 97.16% and 93.93% of the aa residues of the modeled SjGT and SjNCSTN proteins were located in the most favored regions, 0.35% and 1.98% in allowed regions, and only 0% and 1.55% in disallowed regions, respectively (Figure 1C,D). Collectively, these findings indicate that the SjGT and SjNCSTN models had a correct topology with high expected quality.

### 3.5. Bioinformatics Analysis of SjGT and SjNCSTN

**Figure 2:**
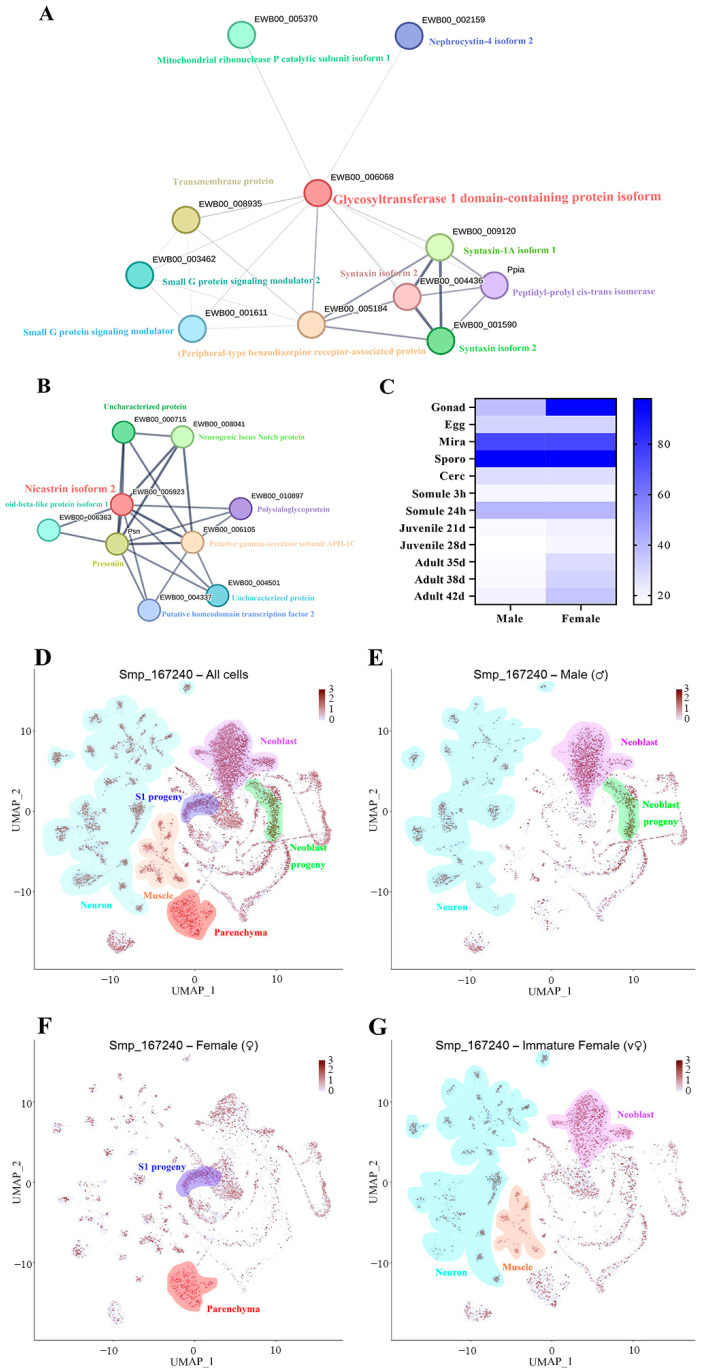
Bioinformatics analysis of SjGT and SjNCSTN. (**A**) Interaction networks of SjGT. (**B**) Interaction networks of SjNCSTN. (**C**) Expression profile of the NCSTN gene in developmental stages of *S. mansoni*. (**D**) Single-cell expression profile of NCSTN gene in all cells of *S. mansoni*. (**E**) Single-cell expression profile of NCSTN gene in different tissues of male, (**F**) female, (**G**) immature female *S. mansoni*.

Protein interaction networks of SjGT and SjNCSTN revealed that the small G protein signaling modulator1/2 transmembrane protein was related to SjGT (Figure 2A). The putative gamma-secretase subunit APH-1C and presenilin were found to interact with SjNCSTN (Figure 2B). These three proteins have been implicated in the formation of amyloid beta peptides, the notch domain, and the notch signaling pathway.

Since no RNA-seq or single-cell related database is available for *S. japonicum*, the *S. mansoni* databases were used as references. The mRNA expression levels of NCSTN at different life stages of mixed-sex infection of *S. mansoni* were analyzed in reference to the RNA seq database (Figure 2C,D) [22]. The results showed that NCSTN mRNA expression was significantly higher in the gonadal, miracidium, and sporocyst stages of females. Meanwhile, NCSTN mRNA expression was significantly up-regulated in the miracidium and sporocyst stages of males. In addition, the single-cell sequencing data showed that NCSTN was mainly expressed in the neuron, neoblast, and neoblast progeny clusters of male *S. mansoni* (Figure 2E); the S1 progeny and parenchyma clusters of mature female *S. mansoni* (Figure 2F); and the neurons, muscles, and neoblasts of immature female *S. mansoni* (Figure 2G). The expression profiles and single-cell expression profiles of GT were not available in the *S. mansoni* database.

### 3.6. Expression and Purification of SjGT and SjNCSTN

The protein-coding sequence regions of the SjGT and SjNCSTN genes were amplified using *S. japonicum* cDNA as a template. Electrophoresis of the samples resulted in two bands of 700 and 550 bp, respectively, as expected. The cDNA products were cloned into the pGEM T-easy vector and sequenced. After double enzymatic cleavage of the products, the SjGT and SjNCSTN genes were cloned into the plasmid pET28a(+) and expressed in *Escherichia coli* BL21 (DE3) cells. The molecular weight of the His-tagged rSjGT and rSjNCSTN proteins were about 29 and 25 kDa, respectively, as confirmed by electrophoresis, with optimal induction times after the addition of isopropyl-b-d-thiogalac-topyranoside of 2 and 4 h, respectively. The lysate was separated into soluble and insoluble fractions (Supplementary Figure S4A,B). Most of the recombinant protein was contained in the supernatant. The proteins were purified by affinity chromatography using His-binding columns under native conditions with dialysis against PBS. The purified rSjGT and rSjNCSTN proteins were separated by electrophoresis and stained with Coomassie brilliant blue (Supplementary Figure S4C,D). A Western blotting analysis further demonstrated that rSjGT and rSjNCSTN could be recognized by antibodies raised against adult worm proteins, highlighting their good utility as immunogens (Supplementary Figure S5).

### 3.7. Immunization with rSjGT and rSjNCSTN Induced Production of IgG-Specific Antibodies

**Figure 3:**
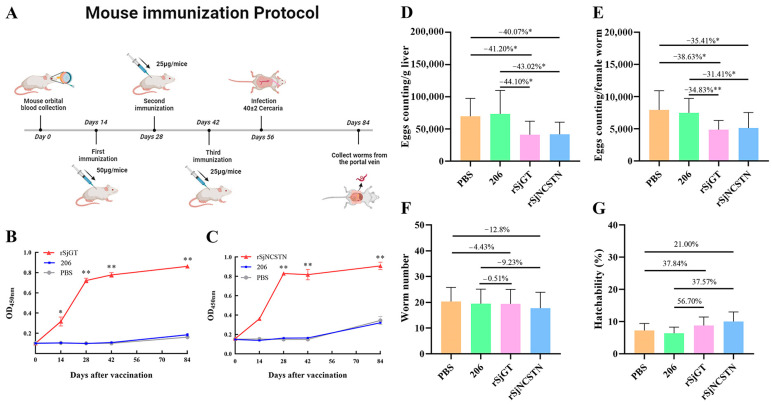
Production of IgG-specific antibodies and evaluation of immunized effects of rSjGT and rSjNCSTN. (**A**) Mouse immunization protocol. (**B**) Specific IgG antibody level analysis for SjGT (n = 10). (**C**) Specific IgG antibody level analysis for SjNCSTN (n = 10). (**D**) Number of liver eggs per gram (n = 10). (**E**) Number of liver eggs per female (n = 10). (**F**) Number of adult worms (n = 10). (**G**) Hatchability of eggs (n = 10). “*” represents the significant difference (*p* < 0.05). “**” represents the significant difference (*p* < 0.01).

Following the administration of a specific immunization protocol (Figure 3A), the serum levels of mouse-specific IgG in all groups were quantified using an ELISA. The serum levels of the IgG antibody were significantly increased in the rSjGT- and rSjNCSTN-immunization groups after the first immunization as compared to the 206-adjuvant and PBS groups; they were further increased after the second immunization and remained at high levels after the third immunization. The IgG antibody levels were significantly higher in the rSjGT- and rSjNCSTN-immunization groups than the PBS and 206-adjuvant groups (Figure 3B,C).

### 3.8. Immunoprotective Effects of rSjGT and rSjNCSTN

The immunoprotective effects of rSjGT and rSjNCSTN were evaluated by calculating the worm and egg burden reduction rates in addition to egg hatchability. Compared with mice in the PBS group and 206-adjuvant group, mice in the rSjGT-immunized group reduced the fecundity of female worms by 38.63% (*p* < 0.05) and 34.83% (*p* < 0.01), and decreased the egg burden of the host liver by 41.2% (*p* < 0.05) and 44.1% (*p* < 0.05), respectively. (Figure 3D,E). In comparison to the PBS and 206 adjuvant groups, female worms in the rSjNCSTN group declined fecundity by 35.41% (*p* < 0.05) and 31.41% (*p* < 0.05), respectively. Additionally, the liver egg burden in the mice from rSjNCSTN group declined by 40.07% (*p* < 0.05) and 43.02% (*p* < 0.05), respectively. (Figure 3D,E). Neither recombinant protein had a significant effect on the adult worm count (Figure 3F) or egg hatchability rate (Figure 3G).

## 4. Discussion

Due to growing concerns about drug resistance associated with the continued use of praziquantel, there is an urgent need for a vaccine to protect against schistosomiasis. Many previous vaccine candidates have been directly targeted to surface proteins. For example, tetraspanin (TSP), a structural protein of the outer tegument, has been investigated as a potential vaccine candidate against schistosomiasis. Immunization of mice with TSP-2 resulted in a 57% reduction in worm burden and a 64% reduction in egg burden in the liver [26]. Similarly, Sm-p80, the large subunit of the calcium-activated neutral protease calpain of *S. mansoni*, plays a crucial role in tegument formation and renewal [27]. When used as a vaccine candidate, rSm-p80 has been shown to reduce egg transmission, inhibit egg hatching, eliminate adult schistosomes, and significantly decrease egg retention in tissues [27,28,29]. In addition, the *S. mansoni* tegument protein Sm29 was shown to protect against parasitic infection by reducing the number of adult worms, intestinal eggs, and liver granulomas by 51%, 60%, and 50%, respectively [28,30]. With the advancement of high-throughput technologies, an increasing number of vaccine candidates are being identified through the screening of differentially expressed proteins in omics studies. In transcriptomics, Smith et al. [31] identified a cloned cDNA corresponding to the 26 kDa glutathione transferase of *S. japonicum* using a lambda gt11 amp3 expression library. The recombinant 26 kDa glutathione transferase was shown to prevent reinfection in pigs and Chinese water buffalo infected with *S. japonicum* by significantly reducing worm and egg burdens in host tissues as well as decreasing egg hatchability [32,33]. In proteomics, heat shock protein 70 was identified as an excretory/secretory protein of *S. japonicum* [34,35]. Recombinant heat shock protein 70 was found to stimulate the expression of several anti-inflammatory factors in mice challenged with cercariae, highlighting its potential as an immunogenic candidate [35]. More recently, our team identified *S. japonicum* translationally controlled tumor protein as a differentially expressed protein pivotal to the growth and development of female worms, particularly during the 18–25 days post-infection period [36]. Subsequent studies demonstrated that recombinant translationally controlled tumor protein significantly reduced the average egg weight per gram of liver tissue and the egg hatching rate of females by 57.94% and 43.16%, respectively [36]. These findings underscore the value of omics and high-throughput data analysis in identifying differentially expressed proteins, providing a robust framework for screening potential vaccine candidates against schistosomiasis.

Our group previously predicted that the SjGT and SjNCSTN proteins might play a role in the development of male *S. japonicum* worms. Although the specific signaling pathways involved in their regulation of reproductive development remain unclear, this study analyzed the fundamental characteristics of these two proteins—such as their signal peptide regions, structural domains, tertiary structures, and potential interaction networks—as well as the expression profiles of their homologs in *S. mansoni*. These analyses may provide a foundational basis for future research on these proteins. Therefore, given their potential impact on schistosome reproductive development, we hypothesize that SjGT and SjNCSTN could serve as promising vaccine candidates.

To evaluate the suitability of SjGT and SjNCSTN as vaccine candidates, their recombinant forms were tested in BALB/c mice. The results demonstrated that both proteins were immunogenic, eliciting a significant production of IgG-specific antibodies. Additionally, immunization with rSjGT and rSjNCSTN was shown to reduce the fecundity of female worms, although neither protein had a notable effect on egg hatchability or the survival of adult worms. Notably, rSjGT appeared to be slightly more effective than rSjNCSTN, potentially due to its role in glycoprotein formation, which is critical for regulating the development and reproduction of helminth. Interestingly, native glycoproteins are known to provide superior immune protection compared to recombinant proteins. This is attributed to their preserved natural conformation and specific structural motifs, which allow precise binding to antigenic determinants and result in the production of high IgG titers, conferring specific passive immunity [37,38]. For instance, studies have shown that native glycoproteins purified using concanavalin A from *Haemonchus contortus* exhibit significantly higher immunoprotective efficacy than their recombinant counterparts [39]. Moreover, the commercial vaccine Barbervax, which incorporates concanavalin A-purified H11 glycoproteins, has been successfully employed to prevent the reproduction of *H. contortus* in ruminants. The N-glycan moieties of the intestinal membrane protein H11, a dominant immunogen in *H. contortus*, demonstrated immunization efficiencies of approximately 75–95% [37,40]. These findings imply that preserving the natural conformation of glycoproteins, such as SjGT, as a protein antigen may enhance its immunogenicity and improve vaccine efficacy. Another protein investigated in this study, SjNCSTN, appears to have distinct functions. Despite our previous findings showing that knockdown of SjNCSTN significantly reduced the reproductive capacity of mature females [10], the present study revealed that SjNCSTN failed to exhibit significant immunoprotective effects. This could be attributed to limitations in the distribution or biological functionality of its recombinant form. Further research is required to optimize its vaccine potential.

To optimize the immunization strategy, future studies could explore the use of combined vaccines. For example, combined vaccines like the diphtheria-tetanus-pertussis and measles-mumps-rubella vaccines have been highly effective in preventing disease. Similarly, co-immunization with a DNA vaccine composed of *S. japonicum* triose-phosphate isomerase fused to heat shock protein 70 and interleukin-12 has been shown to induce protective immunity in water buffalo, achieving a worm reduction rate of 51.2%, a fecal egg reduction of 52.1%, and a liver egg reduction of 61.5% [41]. Furthermore, co-immunization of mice with Sm14 and Sm29 antigens significantly reduced *S. mansoni* infections [42,43]. This enhanced protection may result from the activation of multiple antigen-specific T and B cell clones, thereby eliciting a broader and more robust immune response.

Future vaccine development against schistosomes should focus on optimizing the immunogenicity and efficacy of candidate antigens like glycosyltransferase and nicastrin. Recombinant proteins, in particular, offer several advantages in this regard. They can be produced in large quantities with high purity using standardized bacterial, yeast, or mammalian expression systems, ensuring consistency and scalability for clinical applications [44]. These proteins allow for precise targeting of immunogenic regions, enabling the design of vaccines that elicit specific and robust immune responses while minimizing adverse reactions [45]. Moreover, recombinant protein vaccines are inherently safer, as they eliminate the risk of introducing live parasites or their genetic material into the host [44,45]. With advancements in engineering, recombinant proteins can be enhanced for stability, immunogenicity, and compatibility with adjuvant systems, making them highly versatile candidates for both single and combination vaccine strategies.

Building on these strengths, future efforts should explore combination vaccine strate-gies that integrate multiple antigens targeting various stages of the parasite’s lifecycle, thereby eliciting broader and more robust immune responses. Advances in delivery sys-tems, such as nanoparticle-based formulations or adjuvants tailored to enhance anti-gen-specific immunity, could further improve vaccine efficacy [46]. Future studies should also include large-scale animal models or human trials to validate the effectiveness of these candidates, ensuring scalability and practical application in endemic regions [47]. Notably, the zoonotic transmission of schistosomes highlights the importance of targeting domestic animals, particularly bovines, which are major reservoirs [48]. Vaccinating livestock, alongside human chemotherapy, could significantly reduce environmental egg contamination and transmission rates, aligning with the One Health approach [49].

## 5. Conclusions

In this study, the secondary structures, structural domains, tertiary structures, and potential protein interaction networks of two proteins, SjGT and SjNCSTN, were predicted using bioinformatics tools. Additionally, the immunoprotective effects of their recombinant forms, rSjGT and rSjNCSTN, were evaluated. These findings suggest the potential of SjGT and SjNCSTN as promising vaccine candidates for schistosomiasis, providing a valuable foundation for the development of effective immunization strategies against this parasitic disease.

## Data Availability

The original contributions presented in the study are included in the article. Further inquiries can be directed to the corresponding authors.

## Supplementary Materials

The following supporting information can be downloaded at: https://www.mdpi.com/article/10.3390/pathogens14010070/s1, Figure S1: In silico characterization of SjGT and SjNCSTN. (A) Distribution of signaling peptides of SjGT. (B) Distribution of signaling peptides of SjNCSTN. The signaling peptide located from 1 aa to 23 aa. (C) Distribution of transmembrane regions of SjGT. (D) Distribution of transmembrane regions of SjNCSTN. The transmembrane region located from 658 aa to 680 aa; Figure S2: Homology analysis of SjGT and SjNCSTN. (A) Homology analysis of SjGT. (B) Homology analysis of SjNCSTN; Figure S3: Phylogenetic analysis of SjGT and SjNCSTN. (A) Phylogenetic analysis of SjGT. (B) Phylogenetic trees of SjNCSTN; Figure S4: SDS-PAGE analysis of the expression of recombinant SjGT and SjNCSTN protein. (A) Lane M: protein marker; Lanes 1, 2, 3, 4 and 5 are 0 h, 2 h, 4 h, 6 h, 8 h of rSjGT induction, respectively. (B) Lane M: protein marker; Lanes 1, 2, 3, 4 and 5 are 0 h, 2 h, 4 h, 6 h, 8 h of rSjNCSTN induction, respectively. (C) SDS-PAGE detection of recombinant SjGT protein (Coomassie). Lane M: protein marker; Lane 1: rSjGT induced precipitation; Lane 2: rSjGT induced supernatant; Lane 3: rSjGT recombinant protein purified product. (D) SDS-PAGE detection of recombinant SjNCSTN protein (Coomassie). Lane M: protein marker; Lane 1: SjNCSTN induced supernatant; Lane 2: SjNCSTN induced precipitation; Lane 3: rSjNCSTN recombinant protein purified product; Figure S5: Western blot analysis of recombinant SjGT and SjNCSTN proteins. (A) Western blot of recombinant SjGT protein. Lane M: protein marker; Lane 1 and Lane 2: duplicate samples of purified recombinant SjGT protein. (B) Western blot of recombinant SjNCSTN protein. Lane M: protein marker; Lane 1 and Lane 2: duplicate samples of purified recombinant SjNCSTN protein; Table S1: Primer sequences of SjGT and SjNCSTN gene.
