# Supplementary material for: Antibody Responses and the Vaccine Efficacy of Recombinant Glycosyltransferase and Nicastrin Against Schistosoma japonicum
Source: Pathogens. 2025 Jan 14;14(1):70. doi: 10.3390/pathogens14010070 (PMC11768875; doi:10.3390/pathogens14010070)
Supplement: Supplementary file 1 [file pathogens-14-00070-s001.zip › Supplementary figure and figure legends.pdf]

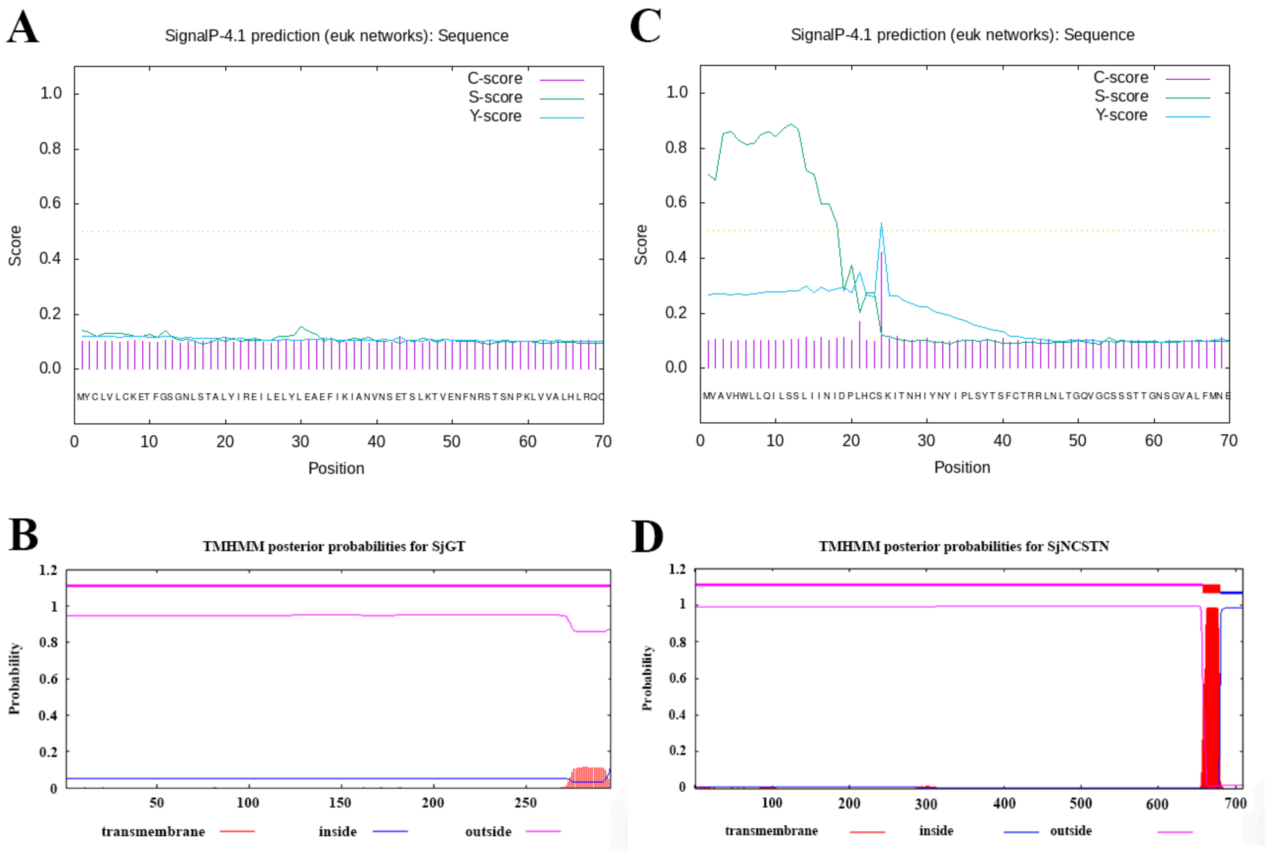

Supplementary Figure S1 ***In silico*** **characterization of SjGT and SjNCSTN.** (A) Distribution of signaling peptides of SjGT. (B) Distribution of signaling peptides of SjNCSTN. The signaling peptide located from 1 aa to 23 aa. (C) Distribution of transmembrane regions of SjGT. (D) Distribution of transmembrane regions of SjNCSTN. The transmembrane region located from 658 aa to 680 aa.

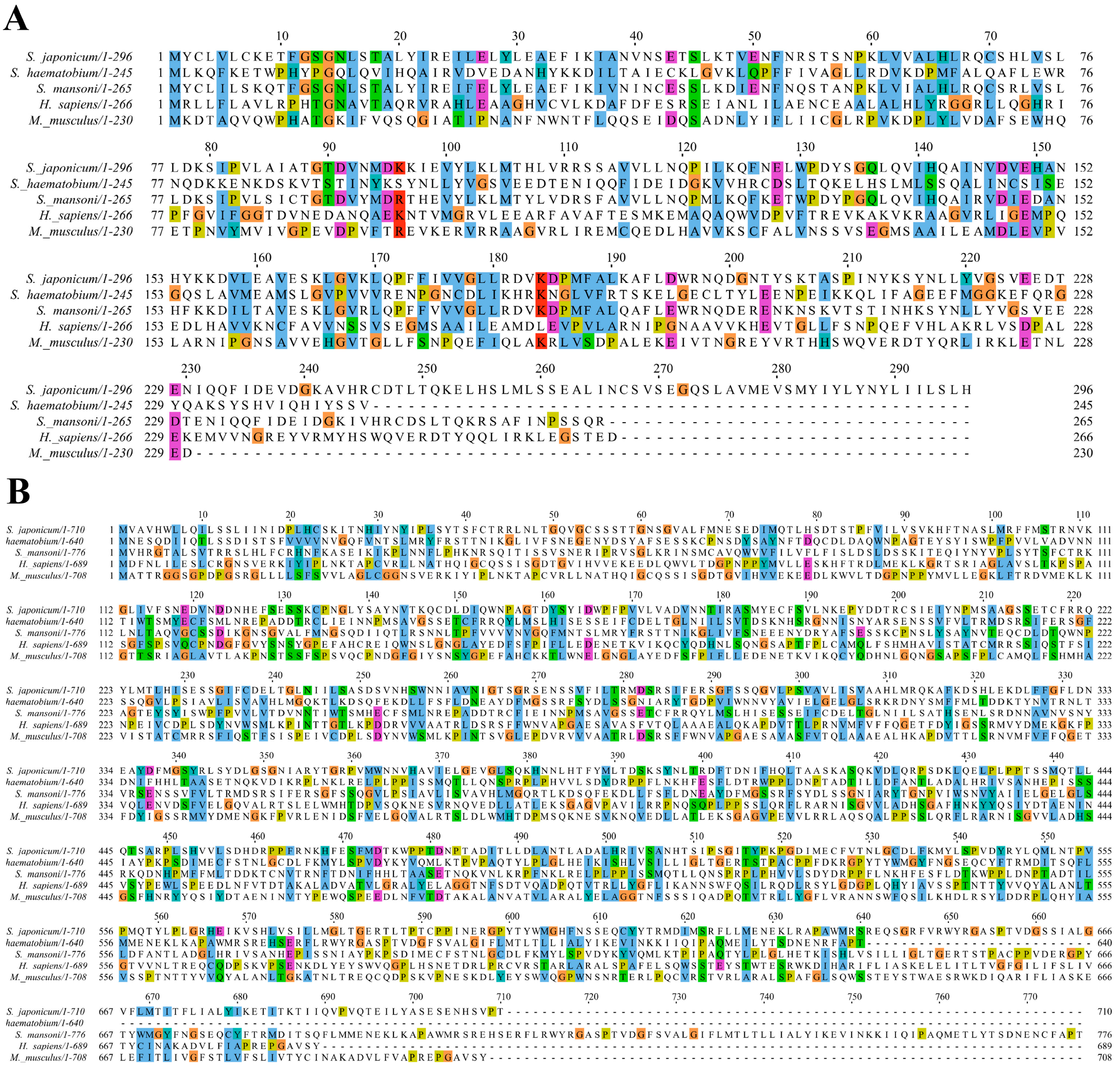

Supplementary Figure S2 Homology analysis of SjGT and SjNCSTN. (A) Homology analysis of SjGT. (B) Homology analysis of SjNCSTN.

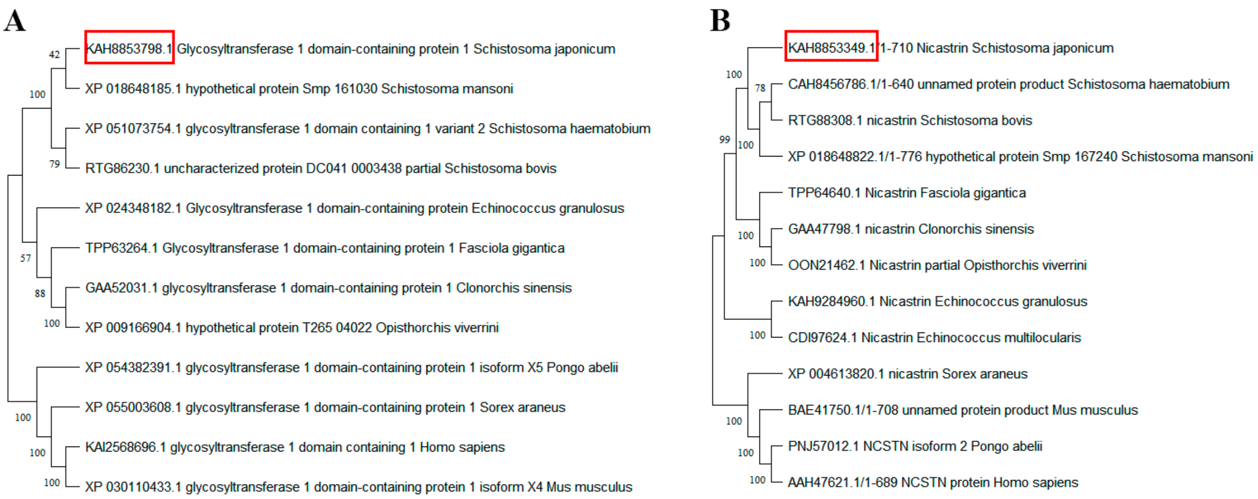

Supplementary Figure S3 Phylogenetic analysis of SjGT and SjNCSTN. (A) Phylogenetic analysis of SjGT. (B) Phylogenetic trees of SjNCSTN.

Protein optimal induction time point screening of rSjGT (left) and rSjNCSTN (right)

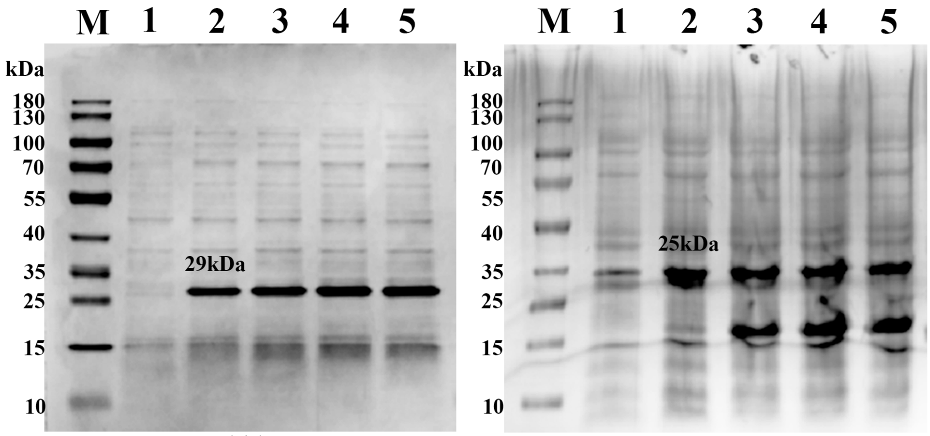

(A) (B)

Protein expression and purification of rSjGT (left) and rSjNCSTN (right)

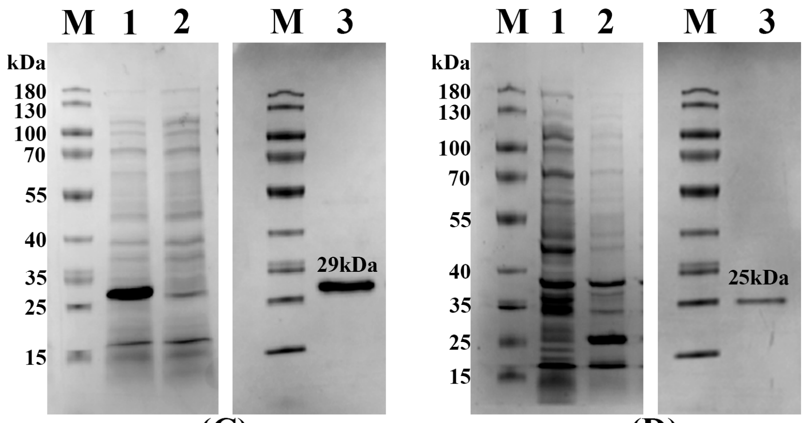

(C) (D)

Supplementary Figure S4 SDS-PAGE analysis of the expression of recombinant SjGT and SjNCSTN protein. (A) Lane M: protein marker; Lanes 1, 2, 3, 4 and 5 are 0 h, 2 h, 4 h, 6 h, 8 h of rSjGT induction, respectively. (B) Lane M: protein marker; Lanes 1, 2, 3, 4 and 5 are 0 h, 2 h, 4 h, 6 h, 8 h of rSjNCSTN induction, respectively. (C) SDS-PAGE detection of recombinant SjGT protein (Coomassie). Lane M: protein marker; Lane 1: rSjGT induced precipitation; Lane 2: rSjGT induced supernatant; Lane 3: rSjGT recombinant protein purified product. (D) SDS-PAGE detection of recombinant SjNCSTN protein (Coomassie). Lane M: protein marker; Lane 1: SjNCSTN induced supernatant; Lane 2: SjNCSTN induced precipitation; Lane 3: rSjNCSTN recombinant protein purified product.

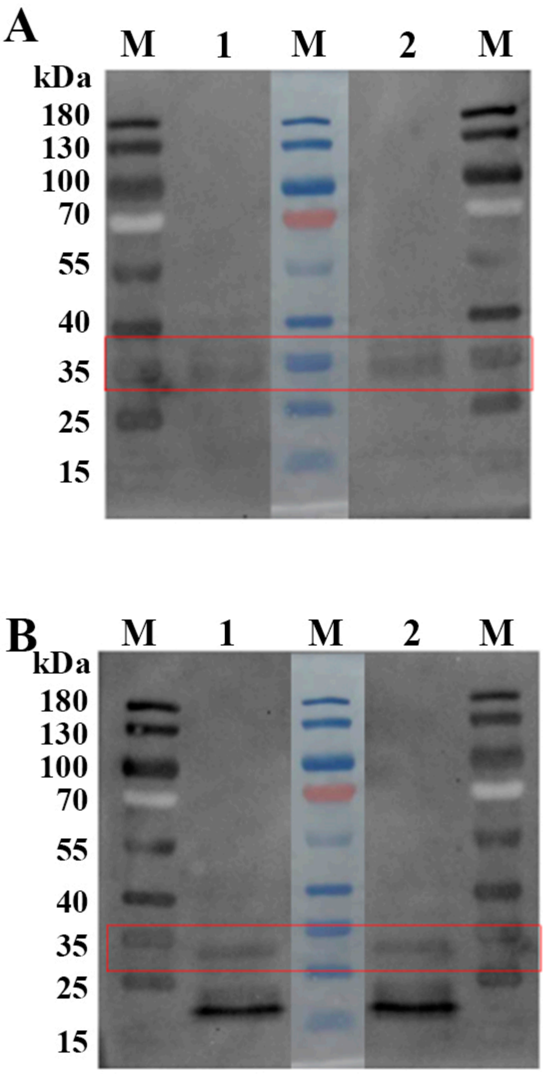

Supplementary Figure S5: Western blot analysis of recombinant SjGT and SjNCSTN proteins. (A) Western blot of recombinant SjGT protein. Lane M: protein marker; Lane 1 and Lane 2: duplicate samples of pu-rified recombinant SjGT protein. (B) Western blot of recombinant SjNCSTN protein. Lane M: protein marker; Lane 1 and Lane 2: duplicate samples of purified recombinant SjNCSTN protein
