# Supplementary material for: Antibody Responses and the Vaccine Efficacy of Recombinant Glycosyltransferase and Nicastrin Against Schistosoma japonicum
Source: Pathogens. 2025 Jan 14;14(1):70. doi: 10.3390/pathogens14010070 (PMC11768875; doi:10.3390/pathogens14010070)
Supplement: Supplementary file 1 [file pathogens-14-00070-s001.zip › Supplementary table S1.docx]

Supplementary Table S1 Primer sequences of *SjGT* and *SjNCSTN* gene

| Gene name | Primer sequences | Restriction enzyme sites (Underlined) |
| --- | --- | --- |
| SjGT | F: 5'-GCTGAATTCTTCCTGCGCATTTCAATAATG-'3 | *Eco*RI |
|  | R: 5'-ATTCTCGAGCTTCTTCAAACTGATCCAAC-'3 | XhoI |
| SjNCSTN | F: 5'-ATAGGATCCATGGAATGTTTTGTAACTA-'3 | *Bam*HI |
|  | R: 5'-ATTCTCGAGTTACGTTGGCACAGA-'3 | XhoI |
