# Supplementary figures and images for: Antibody Responses and the Vaccine Efficacy of Recombinant Glycosyltransferase and Nicastrin Against Schistosoma japonicum
Source: Pathogens. 2025 Jan 14;14(1):70. doi: 10.3390/pathogens14010070 (PMC11768875; doi:10.3390/pathogens14010070)

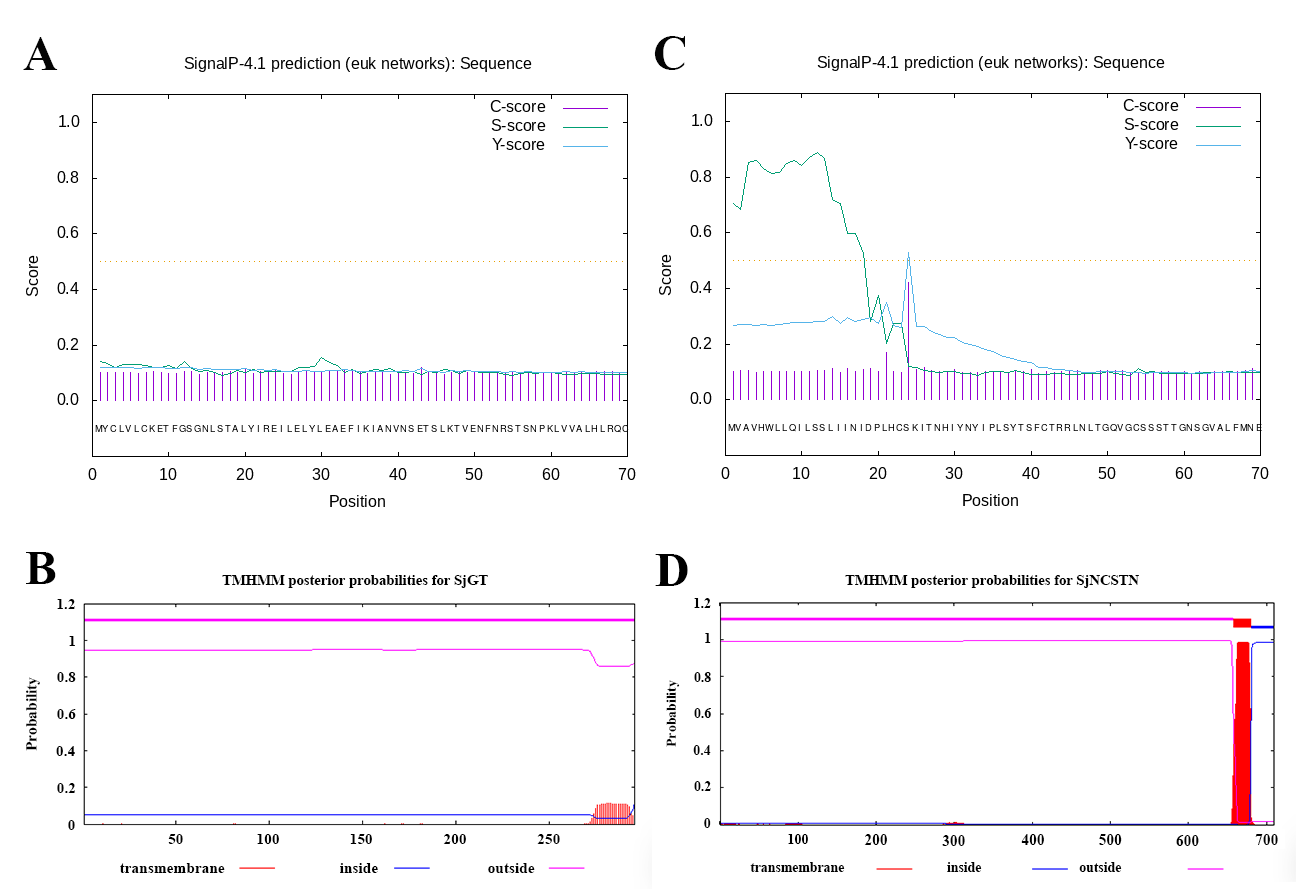

Supplement: Supplementary file 1 [file pathogens-14-00070-s001.zip › Supplementary Figure S1.tif]

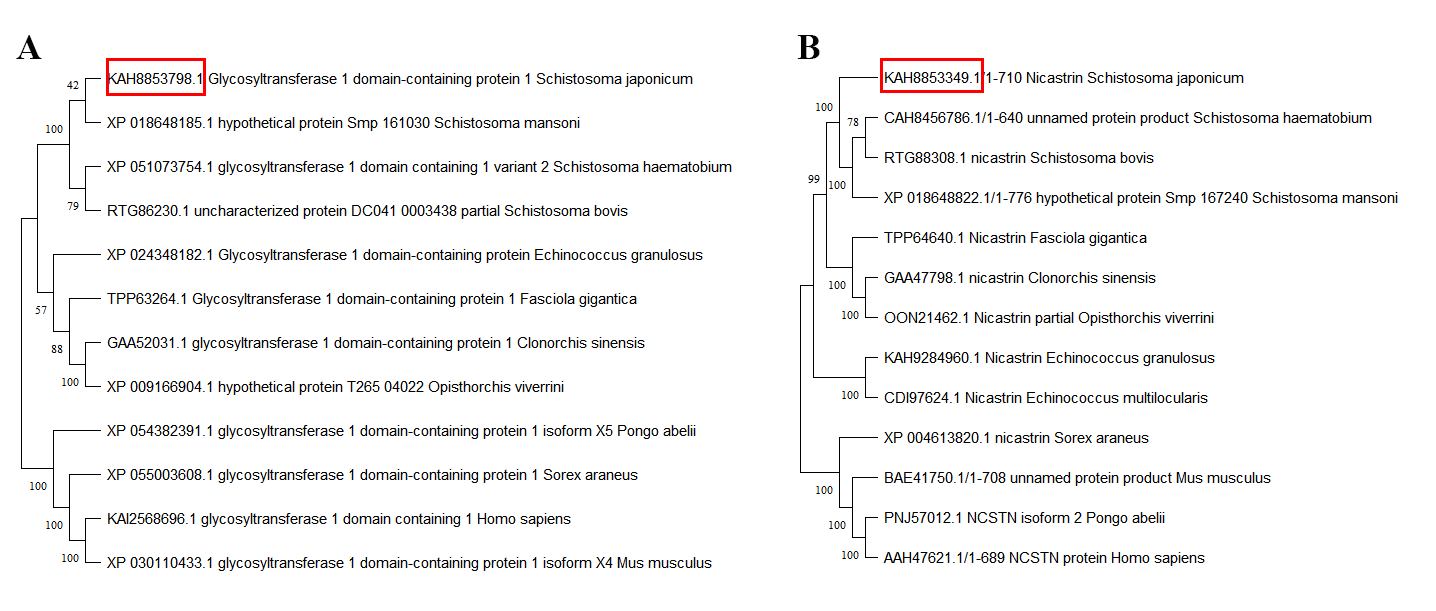

Supplement: Supplementary file 1 [file pathogens-14-00070-s001.zip › Supplementary Figure S3.tif]

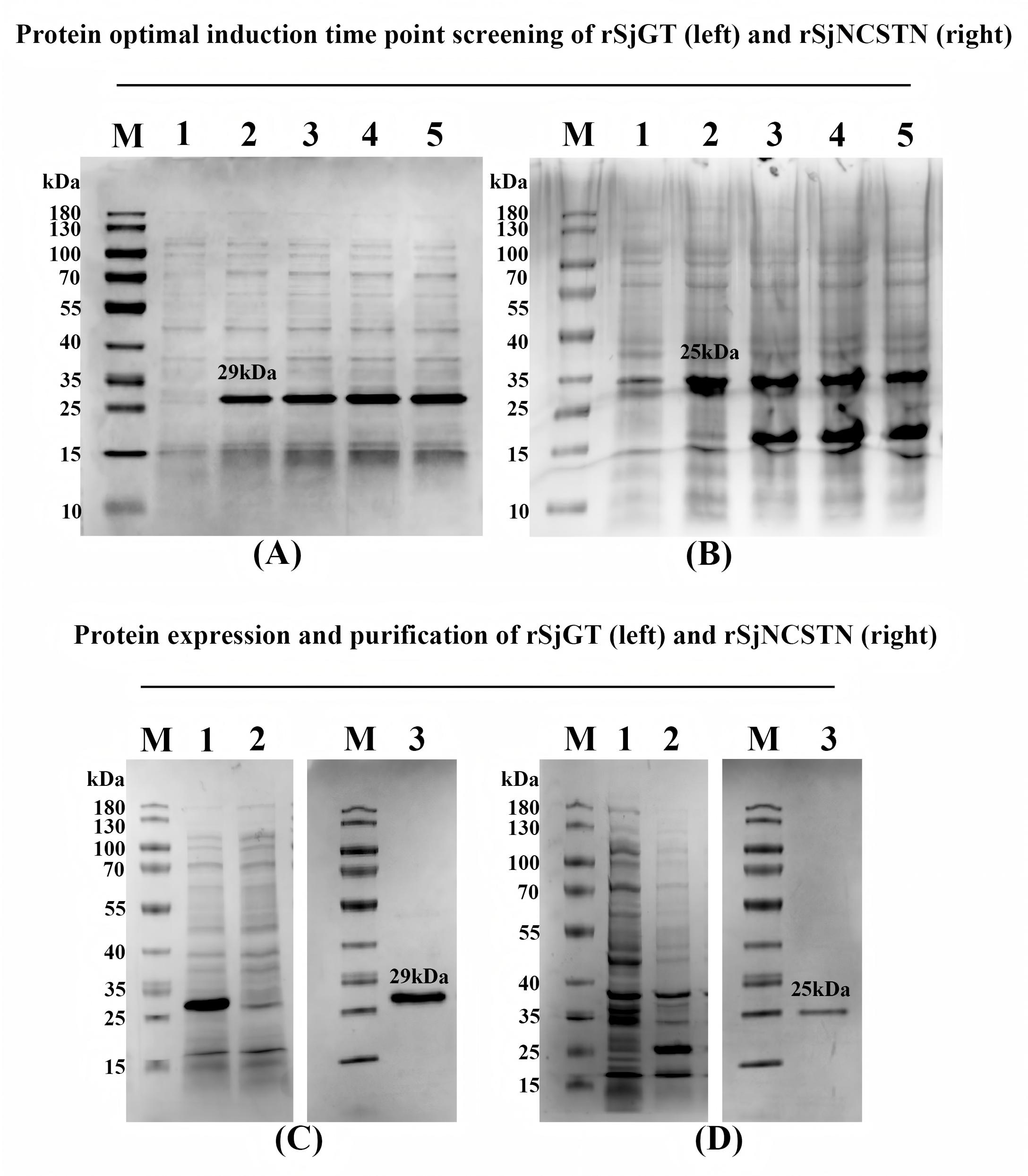

Supplement: Supplementary file 1 [file pathogens-14-00070-s001.zip › Supplementary Figure S4.jpg]

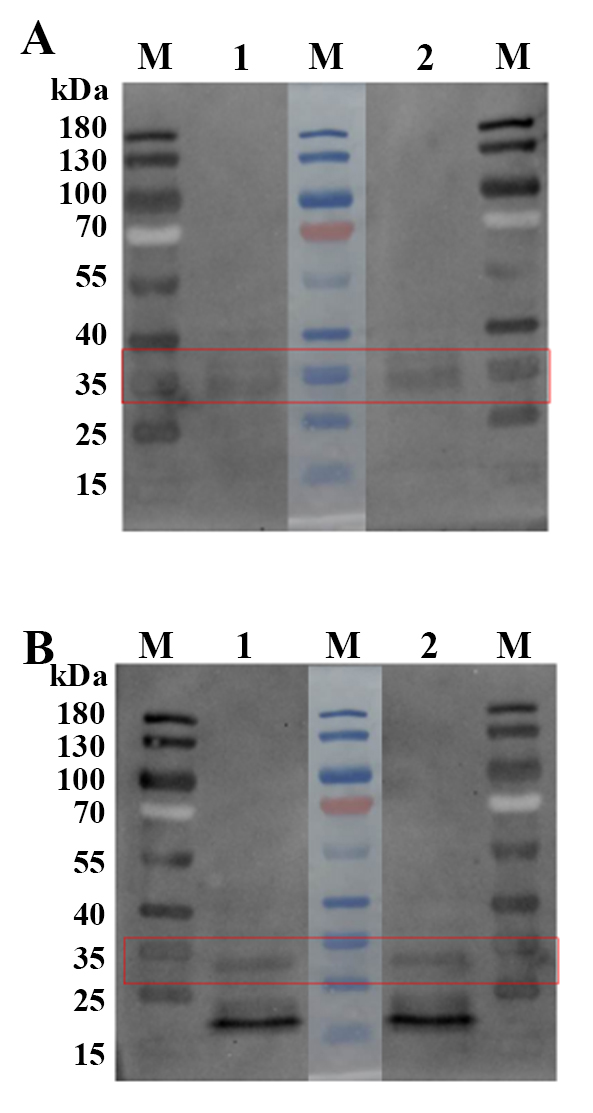

Supplement: Supplementary file 1 [file pathogens-14-00070-s001.zip › Supplementary figure S5.jpg]
